# A Novel Oncolytic Virus Formulation Based on Mesenchymal Stem Cell-Derived Vesicles for Tumor Therapy

**DOI:** 10.7150/jca.104066

**Published:** 2025-01-01

**Authors:** Fanjun Zeng, Yucheng Huang, Bin Xu, Lintong Yao, Yiqing Zhang, Zhiping Gao, Yingli Luo

**Affiliations:** 1 Department of General Practice, Guangdong Provincial Geriatrics Institute, Guangdong Provincial People's Hospital (Guangdong Academy of Medical Sciences), Southern Medical University, Guangzhou 510080, China.; 2 School of Medicine, South China University of Technology, Department of Thoracic Surgery, Guangdong Provincial People's Hospital (Guangdong Academy of Medical Sciences), Southern Medical University, Guangzhou 510006, China.; 3 School of Biomedical Sciences and Engineering, South China University of Technology, Guangzhou International Campus, Guangzhou 510006, China.; 4 Department of Thoracic Surgery, Guangdong Provincial People's Hospital (Guangdong Academy of Medical Sciences), Southern Medical University, Guangzhou 510080, China.; 5 Wuxi School of Medicine, Jiangnan University, Wuxi, Jiangsu 214122, China.

**Keywords:** oncolytic virus, mesenchymal stem cell, cancer treatment, cytotoxic effects, MSC vesicles

## Abstract

Developing new drug delivery systems is crucial for enhancing the efficacy of oncolytic virus (OV) therapies in cancer treatment. In this study, mesenchymal stem cell (MSC)-derived vesicles and oncolytic viruses are exploited to construct a novel formulation. It has been hypothesized that vesicle-coated OVs could amplify cytotoxic effects through superior internalization by tumor cells. MSC vesicles possess natural tumor homing ability and biocompatibility, which can enhance the targeting, uptake, and therapeutic effects of OVs on tumor cells. Experimental results indicated that this treatment system has increased the apoptosis of tumor cells. Furthermore, flow cytometry analysis demonstrated that the uptake of tumor cells by OVs coated with MSC vesicles soared away compared to uncoated OVs, being 1.5 times than that of the uncoated group. Additionally, the confocal laser scanning microscopy also showed that the fluorescence intensity within tumor cells pretreated with MSC-coated OVs was greater. Meanwhile, propidium iodide (PI) staining revealed that MSC-coated Ovs exposed to tumor cells accelerating the apoptosis of the latter. According to the statistics, the number of dead cells was increased, and the flow cytometry testified that the apoptosis in the MSC-coated OV group was as high as 23.78%. These findings highlight the potential of MSC vesicle-coated OVs in enhancing the delivery and efficacy of oncolytic virus therapy, providing a promising strategy for cancer treatment.

## Introduction

Cancer remains one of the highest morbidity and mortality diseases worldwide, with millions of people diagnosed annually and a significant proportion of which result in death. Despite the advancements that have been achieved in conventional therapies, such as surgical resection, radiotherapy, chemotherapy, and immunotherapy, many challenges still persist^1^-^3^. It has been found that traditional treatments often come with severe side effects, and moreover, in the long run there is the possibility for drug resistance. Therefore, it is urgent to make innovative therapeutic approaches to overcome these limitations and offer more effective and less harmful treatment options.

Oncolytic virotherapy has emerged as a promising alternative to traditional cancer treatments^4^-^7^. OVs are either naturally occurring or genetically engineered viruses that selectively infect and lyse cancer cells while sparing normal cells^8^-^12^. This selective targeting is facilitated by the unique characteristics of tumor cells, such as their rapid proliferation, aberrant signaling pathways, and altered immune responses. Beyond their direct lytic effects, OVs have the capability to stimulate the host's immune system, thereby enhancing the body's natural ability to combat the cancer^13^, ^14^. This dual mechanism of action—direct oncolysis and immune system activation—positions OVs as a potentially transformative approach in oncology. Despite their promise, the clinical application of OVs faces significant barriers. The most common method of OV administration in clinical trials is intratumoral injection^15^, ^16^. While this approach ensures direct delivery to the tumor site, it is severely restricted by several factors. These include the body's innate antiviral responses, which can neutralize the viruses before they reach their target, and the physical barriers within the tumor microenvironment that impede viral penetration and diffusion. Consequently, there is an urgent need to develop novel drug delivery systems that can enhance the delivery and efficacy of OVs. One promising strategy to overcome these limitations involves the use of extracellular vesicles (EVs) derived from MSCs as carriers for OVs. As a cell product, EVs has good biological function and function, which is helpful to improve its application in tumor therapy^17^, ^18^. MSCs are multipotent stromal cells that can differentiate into a variety of cell types. They are known for their natural homing abilities to sites of inflammation and injury, including tumors, making them ideal candidates for targeted delivery systems. Additionally, MSC-derived EVs possess low immunogenicity and high biocompatibility, which further enhances their suitability as drug delivery vehicles^19^-^21^. Extracellular vesicles are small, membrane-bound particles released by cells that play a crucial role in intercellular communication. They can encapsulate and transport various biomolecules, including proteins, lipids, and nucleic acids, to recipient cells^22^-^25^. By exploiting the inherent properties of EVs derived from mesenchymal stem cells, researchers can develop effective drug delivery systems to enhance the targeting, uptake, and therapeutic effects of OVs.

In this study, our team hypothesized that MSC-vesicle-coated OVs would be more internalized by tumor cells than uncoated OVs, thereby enhancing their cytotoxic effects. The paper explores a novel formulation that MSC-derived vesicles are utilized to coat OAs, aiming to improve their targeting, uptake, and therapeutic effects on tumor cells. The rationale behind this approach is to take the advantage of the natural homing ability and biocompatibility of MSC membrane to effectively target the tumor cells and promote cellular uptake of OVs. This coating is assumed to facilitate superior internalization of cancer cells and improve the therapeutic treatment by enhancing the delivery of viral payload. To test this hypothesis, we conducted a series of *in vitro* studies to evaluate the uptake, internalization, and cytotoxicity of both coated and uncoated OVs. In the experiment, we firstly obtained biomimetic vesicles by directly compressing mesenchymal stem cells, which are very similar to natural cellular vesicles. Then, applying advanced liposome extrusion technology, we further optimized these vesicles to stably encapsulate oncolytic adenoviruses, thereby shielding them from the host's immune surveillance and enhancing their tumor-targeting ability. The findings showed that OVs encapsulated with MSC vesicles could be taken up by tumor cells more easily compared to uncoated OVs. This was also further validated by Confocal laser scanning microscopy, which showed greater fluorescence intensity within tumor cells with MSC-coated OVs. Additionally, PI staining showed that the number of dead cells increased in the MSC-coated OV group, and flow cytometry data also demonstrated a higher rate of apoptosis. These results highlight the potential of MSC vesicle-coated OVs to improve the delivery and therapeutic efficacy of oncolytic virus therapy. Therefore, we have developed an innovative drug delivery strategy for OVs by utilizing MSC-derived vesicles as carriers. This approach significantly enhances the *in vitro* tumor therapeutic efficacy of OVs, providing a new path to expand the application of oncolytic viruses and enhance their therapeutic effects against tumors. The integration of MSC-derived vesicles with OVs presents a promising strategy to overcome the existing limitations of oncolytic virus therapy and reshape the prospects for cancer treatment. Future research should focus on optimizing this delivery system and evaluating its *in vivo* efficacy, paving the way for clinical applications.

## Materials and Methods

### Materials

Oncolytic virus was obtained from Professor Wang of the University of Chinese Academy of Sciences. MSCs from ATCC cultured in standard conditions. The Annexin V-FITC/PI Apoptosis Detection Kit was purchased from Huisheng Biosciences. Fetal bovine serum (FBS) was obtained from Meilune Biotech. All other reagents are purchased from sigma and used under the guidance of the instructions.

### Preparation and characterization of Mesenchymal Stem Cell derived vesicles-coated Oncolytic Virus (MSC-EV@OV)

MSCs were cultured in DMEM supplemented with 10% FBS and 1% penicillin-streptomycin. Upon reaching 80%∼90% confluence, cells were harvested by using trypsin-EDTA, and washed twice with cold PBS. The cells were further centrifuged at 1000 g for 30 minutes, resuspended in PBS and stored at -80°C for subsequent usage.

Preparation of MSC-EV@OV: The oncolytic adenovirus was propagated in HEK293 cells. The virus was harvested, purified by centrifugation using a cesium chloride gradient, and dialyzed against PBS to remove cesium chloride. The viral titer was tested by using plaque assay. The purified oncolytic virus was mixed with the isolated MSC, and then extruded through a 5 μm polycarbonate membrane by using an Avanti Mini-Extruder (Avanti Polar Lipids, USA) to form MSC vesicles-coated OVs (MSC-OVs). This process involved membrane removal for 11 times to ensure uniform coating.

Characterization of MSC-EV@OV: The hydrodynamic diameter and zeta potential of the MSC-OVs were measured by using a Zetasizer Nano ZS90 (Malvern Instruments, UK). Samples were diluted in deionized water and analyzed at room temperature. The size distribution and zeta potential values were obtained from three independent measurements. The morphology and structure of the MSC-OVs were examined through transmission electron microscopy. A drop of MSC-OV suspension was placed on a carbon-coated grid and adsorbed for 2 minutes. Remove the excess liquid with filter paper, and then stain the grid with 2% phosphotungstic acid for 1 minute. After drying, the samples were observed under a TEM (JEOL JEM-2100, Japan) at an accelerating voltage of 200 kV. Images were captured to evaluate the shape and coating uniformity of MSC-EV@OV. Images could be captured to assess the shape and coating uniformity of the MSC-EV@OV.

### Cellular uptake of Cy5-labeled OV and MSC-EV@OV

OVs were labeled with Cy5 dye based on the standard NHS-ester labeling protocol. Briefly, purified OVs were incubated with Cy5-NHS ester (v/v=400/1) in PBS at room temperature for 1 hour, followed by dialysis against PBS to remove unbound dye. The Cy5-labeled OVs were coated with MSC vesicles as stated previously. In short, Cy5-labeled OVs were mixed with isolated MSC vesicles in a mass ratio 1:5, and then the mixture was extruded through a 200 nm polycarbonate membrane using an Avanti Mini-Extruder (Avanti Polar Lipids, USA) to form MSC vesicles coated with Cy5-labeled OVs.

Cellular Uptake Studies: B16-F10 melanoma cells were seeded in 12-well plates for flow cytometry analysis and on glass coverslips in 24-well plates for confocal microscopy. To assess the cellular uptake of OV and MSC-EV@OV, B16-F10 cells were incubated with equivalent amounts of Cy5-labeled OV and MSC-EV@OV (108 PFU/mL) for 4 hours at 37°C. Following incubation, cells were washed three times with cold PBS to remove unbound particles and detached using trypsin-EDTA. The cells were resuspended in 500 μL of PBS containing 2% FBS. Flow cytometry analysis was performed using a BD FACSCalibur to measure the fluorescence intensity of Cy5 in the cells. Data were analyzed using FlowJo software.

For confocal microscopy, B16-F10 cells were incubated with Cy5-labeled OV and MSC-EV@OV (108 PFU/mL) for 8 hours at 37°C. After incubation, the cells were washed three times with cold PBS and fixed with 4% paraformaldehyde for 15 minutes at room temperature. Cells were then permeabilized with 0.1% Triton X-100 in PBS for 5 minutes and blocked with 5% BSA in PBS for 1 hour. The cells were stained with DAPI (Sigma-Aldrich, USA) for 5 minutes to visualize the nuclei and mounted on glass slides using Fluoromount-G. Confocal laser scanning microscopy was performed using a Zeiss LSM 710 microscope (Carl Zeiss, Germany) to capture fluorescence images. Images were processed and analyzed using ImageJ software (NIH, USA).

### Cytotoxicity assay of OV and MSC-EV@OV on tumor cells

B16-F10 melanoma cells were seeded at a density of 5 × 103 cells per well in 96-well plates for the CCK-8 assay and at a density of 1 × 105 cells per well in 24-well plates for the PI staining assay.

Cytotoxicity Assay: To evaluate the cytotoxic effects of OV and MSC-EV@OV on B16-F10 cells, the Cell Counting Kit-8 (CCK-8, Dojindo, Japan) assay was performed. After seeding, the cells were allowed to adhere overnight. The next day, cells were treated with 106 PFU/mL of OV and MSC-EV@OV for 24 hours. At each time point, 10 μL of CCK-8 reagent was added to each well, and the cells were incubated for an additional 2 hours at 37°C. The absorbance was measured at 450 nm using a microplate reader (Bio-Rad, USA). Cell viability was calculated as a percentage relative to the untreated control cells. Each experiment was performed in triplicate.

To further assess cell death, PI staining was used to detect dead cells. B16-F10 cells were treated with OV and MSC-EV@OV at the same concentrations mentioned above for 12 hours. Following treatment, the cells were washed twice with cold PBS and incubated with 5 μg/mL PI (Sigma-Aldrich, USA) in PBS for 15 minutes at room temperature in the dark. The cells were then washed with PBS and observed under a fluorescence microscope (Nikon, Japan) to detect PI-positive cells.

### Apoptosis detection of tumor cells treated by OV and MSC-EV@OV

Apoptosis Detection: B16-F10 melanoma cells were seeded at a density of 1 × 105 cells per well in 24-well plates and allowed to adhere overnight. The next day, cells were treated with OV and MSC-EV@OV at concentrations of 106 PFU/mL for 8 hours. To evaluate apoptosis, the Annexin V-FITC/PI apoptosis detection kit (BD Biosciences, USA) was used. After treatment, cells were washed twice with cold PBS and resuspended in 1× binding buffer at a concentration of 1 × 106 cells/mL. 100 μL of the cell suspension was transferred to a 5 mL culture tube, followed by the addition of 5 μL of Annexin V-FITC and 5 μL of PI. The cells were gently vortexed and incubated for 15 minutes at room temperature in the dark. After incubation, 400 μL of 1× binding buffer was added to each tube, and the samples were analyzed by flow cytometry within 1 hour.

Flow cytometry was performed using a BD FACSCalibur (BD Biosciences, USA). Data acquisition and analysis were carried out using FlowJo software. Cells that were Annexin V-FITC positive and PI negative were considered early apoptotic, whereas cells that were positive for both Annexin V-FITC and PI were considered late apoptotic. The percentage of apoptotic cells (both early and late) was calculated and compared between the OV-treated and MSC-EV@OV-treated groups.

## Results and Discussion

### Characterization of OV, MSC-EV, and MSC-EV@OV

**Figure 1:**
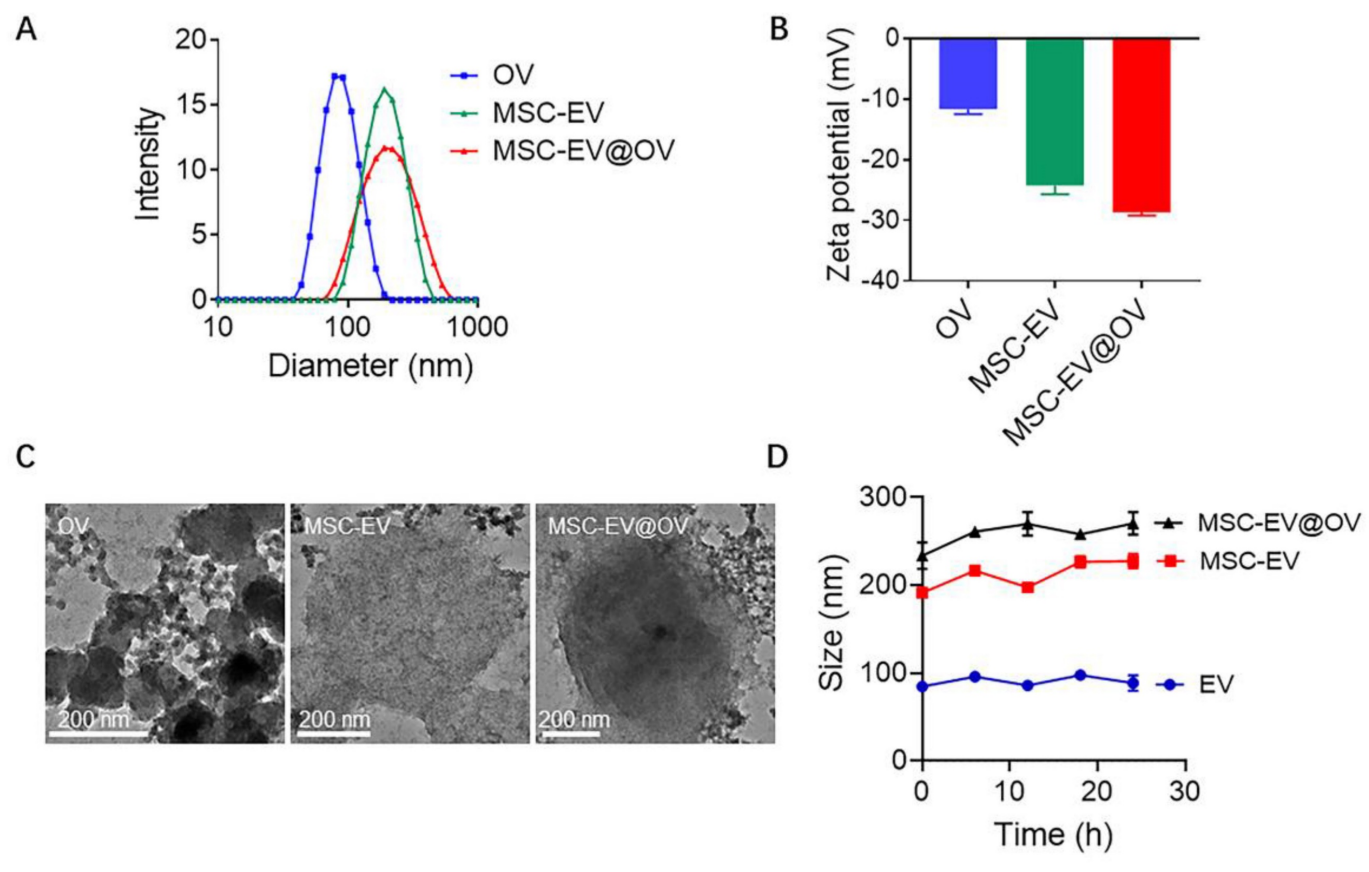
Characterization of OV, MSC-EV and MSC-EV@OV. The hydrodynamic sizes (A) and zeta potential (B) identified by the DLS measurements. (C) TEM images of different OV formulations. (D) Stability assessment of OV, MSC-EV, and MSC-EV@OV over 24 hours *in vitro*.

The physical properties of OV, MSC-EV, and MSC-EV@OV were characterized using dynamic light scattering (DLS) and transmission electron microscopy (TEM). The DLS analysis revealed that the average hydrodynamic diameters of OV, MSC-EV, and MSC-EV@OV were 78.8 nm, 190 nm, and 220 nm, respectively (Figure 1A). These results indicate a significant increase in particle size upon coating OV with MSC-EV, confirming the successful encapsulation of OV within the vesicles. The corresponding zeta potentials were measured to be -11.7 mV for OV, -24.2 mV for MSC-EV, and -28.7 mV for MSC-EV@OV (Figure 1B). The more negative zeta potential of MSC-EV and MSC-EV@OV compared to OV alone suggests an increased surface charge due to the presence of the vesicle membrane, which can enhance stability and prevent aggregation in biological environments.

TEM images in Figure 1C provided further insights into the morphological characteristics of the particles. The OV appeared as uniform spherical particles with consistent size distribution. For MSC-EV and MSC-EV@OV, TEM images showed vesicle structures with a clear contrast difference indicating the presence of a membrane layer around the encapsulated OV. This structural observation supports the successful coating of OV by MSC vesicles. The encapsulation of OV within MSC-EV was further evidenced by the altered contrast of the vesicles. To assess the stability of these formulations, the size of OV, MSC-EV, and MSC-EV@OV was monitored over 24 hours. There were no significant changes in size for any of the particles, indicating good stability of the formulations *in vitro* (Figure 1D). This stability is critical for potential therapeutic applications as it ensures that the nanoparticles maintain their integrity and functional properties during storage and circulation in the body.

### Cellular uptake of OV and MSC-EV@OV

**Figure 2:**
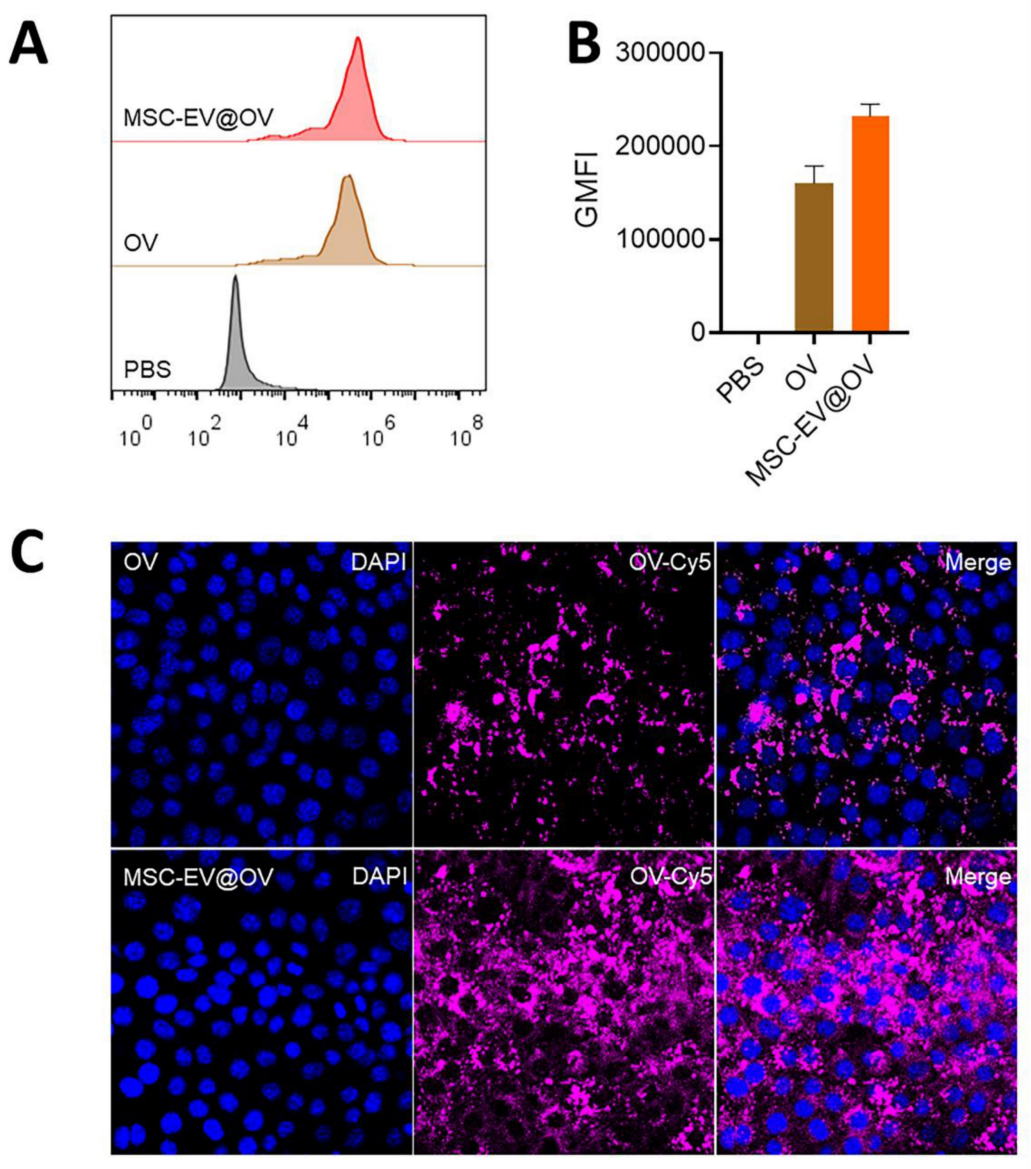
The cellular uptake of different formulations. (A, B) Flow cytometry analysis of tumor cells treated with OV and MSC-EV@OV. (C) Confocal laser scanning microscopy images of fluorescence intensity within tumor cells treated with OV and MSC-EV@OV.

Flow cytometry analysis demonstrated a significant difference in the uptake of OV and MSC-EV@OV by tumor cells. The fluorescence intensity of the virus in the MSC-EV@OV-treated group was 1.5 times higher than that in the OV-treated group, indicating enhanced uptake (Figures 2A and 2B). Confocal laser scanning microscopy further supported these findings, showing stronger fluorescence intensity within tumor cells treated with MSC-EV@OV compared to those treated with OV alone (Figure 2C). This suggests that the MSC-EV coating facilitates better internalization of the virus by tumor cells. Hence, the results indicate that MSC-EV coating significantly enhances the uptake of oncolytic viruses by tumor cells, as evidenced by both flow cytometry and confocal laser scanning microscopy. This increased uptake translates to improved therapeutic efficacy, highlighting the potential of MSC-EV@OV as a superior formulation for cancer treatment. The stability and enhanced internalization underscore the viability of this approach, paving the way for further preclinical and clinical evaluations.

### Cytotoxic effect of OV and MSC-EV@OV on the tumor cells

**Figure 3:**
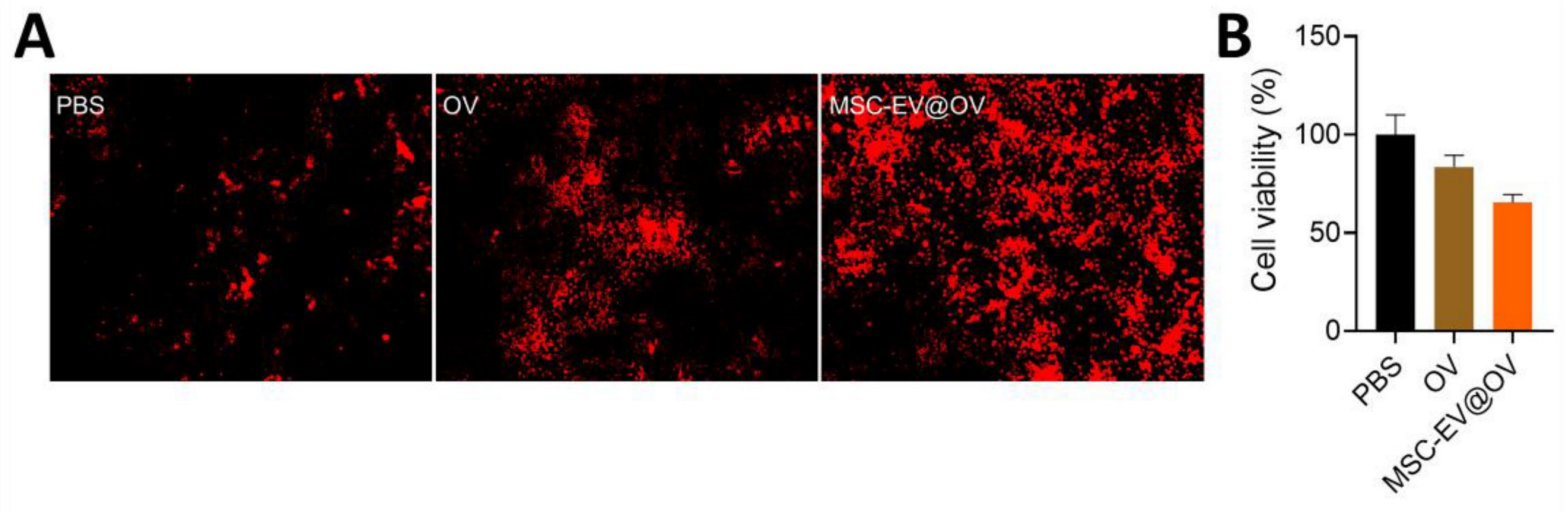
The cellular ability treated with different formulations. (A) PI staining results of red fluorescence in tumor cells treated with OV and MSC-EV@OV. (B) CCK-8 assay results of tumor cell viability following MSC-EV@OV treatment compared to OV treatment.

The therapeutic efficacy of OV and MSC-EV@OV on tumor cells was evaluated using PI staining and CCK-8 assays. PI staining results indicated that MSC-EV@OV-treated tumor cells exhibited more red fluorescence compared to the OV-treated group, reflecting a higher degree of cell death (Figure 3A). This was corroborated by the CCK-8 assay results, which showed a significant reduction in tumor cell viability to 65% following treatment with MSC-EV@OV. In contrast, cells treated with OV alone exhibited a higher survival rate.

The enhanced uptake of MSC-EV@OV by tumor cells, as indicated by flow cytometry and confocal microscopy, likely contributes to the increased cytotoxicity observed. The MSC-EV coating appears to improve the delivery and internalization of the oncolytic virus, leading to more effective tumor cell killing. These findings suggest that the use of MSC-derived vesicles to encapsulate and deliver OVs could be a promising strategy to improve the therapeutic outcomes of oncolytic virotherapy. The stability of the formulations further supports their potential use in clinical settings, where consistent performance over time is crucial.

### Apoptosis of tumor cells treated with OV and MSC-EV@OV

**Figure 4:**
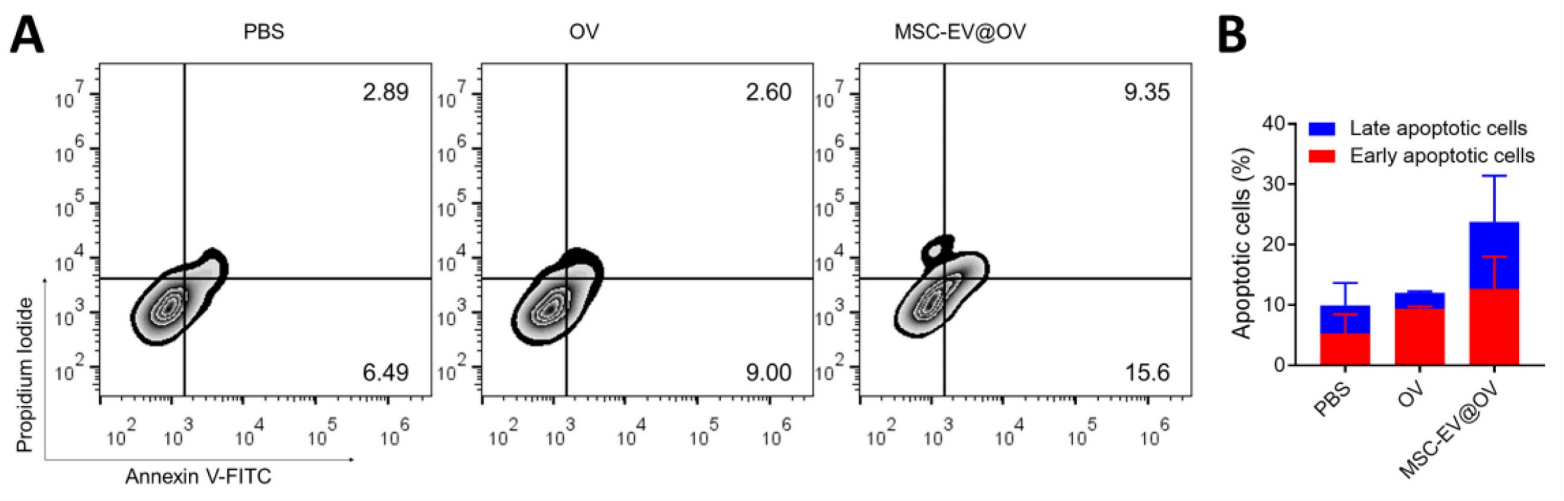
The apoptosis of tumor cells treated by the different formulations. (A) Flow cytometry analysis and (B) statistical analysis of apoptotic tumor cells following the OV and MSC-EV@OV treatment.

Flow cytometry analysis was conducted to assess the apoptotic status of tumor cells following OV and MSC-EV@OV treatment. The results demonstrated a significant increase in both early and late apoptosis compared to the control group. Specifically, early apoptosis was enhanced by 2.5-fold, and late apoptosis by 3.2-fold relative to the control group (Figures 4A and 4B). Overall, the proportion of apoptotic cells was markedly higher in the MSC-EV@OV-treated group compared to PBS and OV alone. The significant increase in apoptotic cell populations in the MSC-EV@OV group compared to PBS and OV alone underscores the therapeutic potential of this combination. Future studies should focus on elucidating the molecular mechanisms underlying this enhanced apoptotic response and evaluating the *in vivo* efficacy of MSC-EV@OV treatment in various tumor models.

## Conclusion

This study highlights the potential of MSC-derived exosomes as a new drug delivery system to enhance the efficacy of OV therapy. Our findings indicate that MSC vesicle-coated OVs significantly improve targeting, uptake, and therapeutic efficacy against tumor cells compared to those of uncoated counterparts. Leveraging the intrinsic homing capability and biocompatibility of MSC vesicles, our approach effectively enhances the internalization of OVs by tumor cells and yields superior cytotoxic effects.

Flow cytometry analysis showed MSC vesicle-coated OVs were internalized by tumor cells about 1.5 times higher than the uncoated group, indicating the enhanced targeting capability. This was further confirmed by confocal laser scanning microscopy, which revealed greater fluorescence intensity observed in tumor cells treated with MSC-coated OVs. The superior internalization of these coated OVs underscores the potential of MSC vesicles to improve the delivery of therapeutic drugs to tumor cells.

Moreover, the therapeutic efficacy of MSC vesicle-coated OVs was significantly enhanced in our apoptosis experiment. PI staining showed that the number of dead cells coated by MSCs in the OV group is higher, indicating the vitality of tumor cells on a decline. Additionally, flow cytometry testified that the rate of apoptosis of tumor cells treated with OVs-coated MSC vesicles was significantly higher than those of uncoated OVs. These findings suggest that MSC vesicle coating not only promotes the superior absorption of OVs by tumor cells, but also strengthens the cytotoxic effect of OVs on tumor cells. These results highlight the potential of MSC vesicle-encapsulated OVs to considerably intensify the delivery and efficacy of oncolytic virus therapy, providing a promising strategy for improving cancer treatment.

Using mesenchymal stem cell-derived vesicles as a delivery system for OVs represents a significant advancement in cancer therapy. However, optimizing the utilization of mesenchymal stem cell-derived vesicles as a delivery system for OVs requires more in-depth research and experimental validation. On the basis of the recent discoveries, the research should explore how MSC vesicles enhance the targeting and uptake of OVs to maximize the therapeutic effect and minimize the off-target effects. Important domains for future exploration include the enhancement of vesicle engineering techniques to improve the loading capacity and stability of OVs, as well as investigations into their biodistribution, tumor penetration, and interactions with the immune system. These initiatives will play a crucial role in surmounting the limitations of existing conventional OV therapies and facilitate the development of more effective and targeted treatment for cancer.

In conclusion, MSC vesicle-coated OVs offer immense promises an innovative approach for enhancing oncolytic virus therapies. The significant improvements in uptake, targeting, and apoptotic effects observed in this study enable the potential of this combined approach to revolutionize the cancer treatment. Ongoing research area should focus on elucidating the mechanisms underlying the enhanced effects of MSC vesicle-coated OVs and exploring their efficacy in various *in vivo* tumor models. The continuous development of this strategy is leading to more effective and targeted cancer therapies, ultimately improving patients with cancer.
